# Clerosterol from vinegar-baked radix bupleuri modifies drug transport

**DOI:** 10.18632/oncotarget.15212

**Published:** 2017-02-09

**Authors:** Ya Zhao, Li-Min Feng, Li-Juan Liu, Xian Zhang, Rui-Zhi Zhao

**Affiliations:** ^1^ Department of Chinese Medicine Property Team, Second Affiliated Hospital, Guangzhou University of Chinese Medicine, Guangzhou, Guangdong, China; ^2^ The Postdoctoral Research Station, Guangzhou University of Chinese Medicine, Guangzhou, Guangdong, China

**Keywords:** clerosterol, transporter, Pgp, Mrp, Oct

## Abstract

Vinegar-baked Radix Bupleuri (VBRB) is reportedly used to treat liver cancer when combined with traditional chemotherapy and data show that this combination may modify drug transport. We isolated clerosterol from VBRB and studied its effect on drug transporters in normal or transporter-overexpressing cells. Transporter activity was assayed using cellular substrate concentration and transporter expression with Western blot and RT-qPCR. Clerosterol decreased cisplatin uptake in BRL cells mainly through increasing Mrp2 gene expression. Clerosterol also decreased the uptake of colchicine in HEK 293 cells by increasing both Pgp and Mrp1 activity; in detail, it could increase Pgp protein but had marginal effects on Mrp1 protein and gene expression. Further study showed clerosterol increased OCT2 activity in HEK293-Pgp cells by increasing OCT2 protein and mRNA. Clerosterol could suppress Pgp overexpression but not by regulating protein and gene expression. And clerosterol had marginal effects on Mrp2 and Mrp1 activity in Mrp2- and Mrp1-overexpressing HEK293 cells. Thus, Clerosterol may be an active constituent of VBRB and may work against cancer multidrug resistance by inhibiting Pgp activity.

## INTRODUCTION

Liver cancer is often fatal, as patients are often diagnosed at late stages of the disease. For those who can be treated, surgical resection is often the first-line treatment, frequently accompanied by chemotherapy [1]. Drugs used to treat hepatic cancers have many side effects and inter-individual therapeutic efficacy, and drug resistance is an increasing problem. Thus, a method for increasing treatment efficacy is desired.

Often, drug combinations offer more therapeutic potential than single agents, and smaller doses may be used of each to reduce side effects. Radix Bupleuri, the dry radix of Bupleurum Chinese DC and Bupleurum scozonerifolium, is a widely applied Traditional Chinese Medicine, used to treat fever and other diseases. It has been used to treat liver-related diseases after being baked with vinegar. Clinically, VBRB with traditional chemotherapy may be promising as a novel treatment for liver cancer [2, 3]. Data show that Xiao Caihu Tang (a VBRB representative) with chemotherapy, such as cyclophosphamide (CTX) or cisplatin, improves the quality of life in animals as well as inhibits cell growth and promotes hepatic tumor apoptosis [4, 5]; however, the effective constituents in VBRB and its mechanism of action are unclear.

Multidrug resistance (MDR) is the main cause of decreasing chemotherapeutic tumoricidal activity. Overexpression of ABC transporters is related to MDR, and the main transporters are P-glycoprotein (P-gp) and multidrug resistance-associated protein (MRPs/Mrps) [6, 7]. Radix Bupleuri was reported to reverse multidrug resistance in liver cancer [8, 9], so modulating transporter expression may explain increasing antitumor efficacy. OCT2/Oct2 is an influx transporter that moves cationic compounds, and it may be an ideal target for reducing ototoxicity and nephrotoxicity of chemotherapeutics [10, 11]. Thus, we studied P-gp, Mrp1, Mrp2, and OCT2 transporters and underlying mechanisms of their modulation.

Our previous work indicated that ethyl acetate and n-butanol fractions were active constituents of VBRB. The n-butanol fraction mainly contains saikosaponins and several investigations about the effect of saikosaponins on liver cancer and mechanisms behind this activity have been published [12–14]; however, the ethyl acetate fraction has not been studied much. Thus, we investigated effective compounds of the ethyl acetate fraction and mechanisms of such activity.

## RESULTS

### Clerosterol characterization

**Figure 1:**
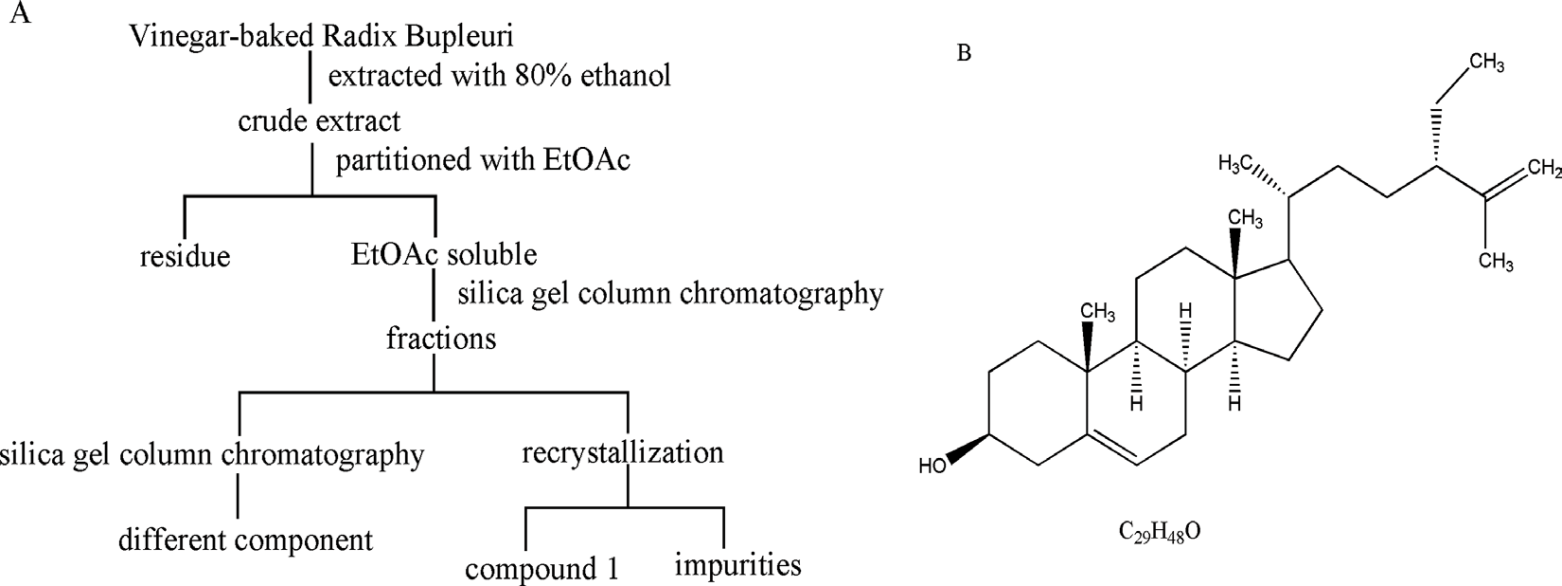
Isolation and purification of compound 1 from VBRB (A) and clerosterol structure (B)

Compound 1 was obtained as a white needle-like crystal. EI-MS showed a quasi-molecular ion at m/z 413 [M-H]^−^, so the molecular formula was assigned as C_29_H_48_O combined with ^13^C NMR data (C × 29). ^1^H NMR and ^13^C NMR spectra showed signals as follows: ^1^H NMR (500 MHz, CDCl_3_): *δ* 5.35 (1H, brd, J = 5.2 Hz, H-6), 4.72 (1H, dd, J = 1.4, 2.5 Hz, H-27b), 4.64 (1H, d, J = 2.5 Hz, H-27a), 3.52 (1H, m, H-3), 2.26 (2H, m, H-4a, H-24), 1.98 (2H, m, H-4b, H-7a), 1.84 (4H, m, H-7b, H-15a, H-16a, H-20), 1.56 (3H, s, H-26), 1.56 (2H, m, H-2a, H-12a), 1.49 (6H, m, H-8, H-9, H-11a, H-17, H-28), 1.29 (7H, m, H-2b, H-11b, H-12b, H-15b, H-16b, H-23), 1.09 (2H, m, H-1b, H-22), 1.00 (3H, s, H-18), 0.90 (3H, d, J = 6.6 Hz, H-21), 0.80 (3H, t, J = 7.4 Hz, H-29), 0.67 (3H, s, H-19). ^13^C NMR (125 MHz, CDCl_3_): *δ* 147.7 (s, C-25), 140.9 (s, C-5), 121.9 (s, C-6), 111.5 (t, C-27), 72.0 (d, C-3), 56.9 (d, C-14), 56.2 (d, C-17), 50.3 (d, C-9), 49.7 (d, C-24), 42.5 (d, C-13), 42.5(t, C-4), 39.9 (t, C-12), 37.4 (t, C-1), 36.7 (s, C-10), 35.7 (d, C-20), 33.8 (t, C-22), 32.1 (t, C-7), 31.8 (d, C-8), 29.5 (t, C-23), 28.3 (t, C-16), 26.7 (t, C-28), 24.4 (t, C-15), 21.2 (t, C-11), 19.6 (q, C-19), 18.8 (q, C-21), 17.9 (q, C-26), 12.2 (q, C-18), and 12.0 (q, C-29). NMR data agreed with the literature [15], so compound 1 was identified as clerosterol (Figure 1B), representing the first isolation from Radix Bupleuri.

### Clerosterol had marginal effects on BRL and HEK293 cell viability

Viability was assessed in BRL and HEK293 cells treated with clerosterol (0.24–243.0 μM) and data showed no inhibitory effects of clerosterol treatment on BRL cells up to 24.3 μM and in HEK293 cells up to 121.5 μM, so these thresholds were used for additional experiments.

### Identification of HEK293-OCT2, HEK293-Mrp2, and HEK293-Pgp stable cell lines

**Figure 2:**
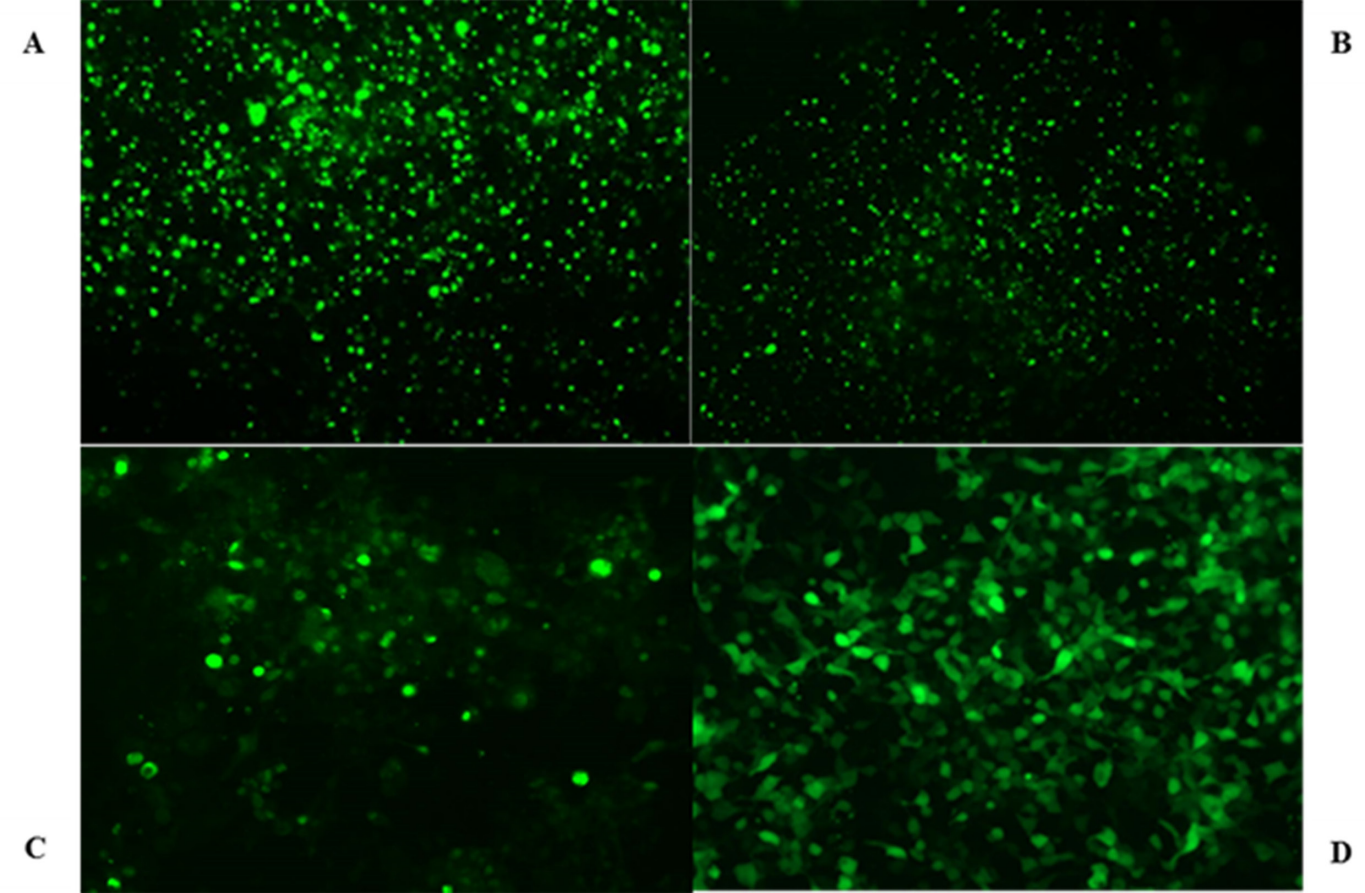
Micrograph of transfected cells HEK293 transfected cells overexpressing OCT2 **(A)**, Mrp2 **(B)**, Pgp **(C)**, and HEK293-vector cells (D) were photographed by fluorescent microscope.

**Figure 3:**
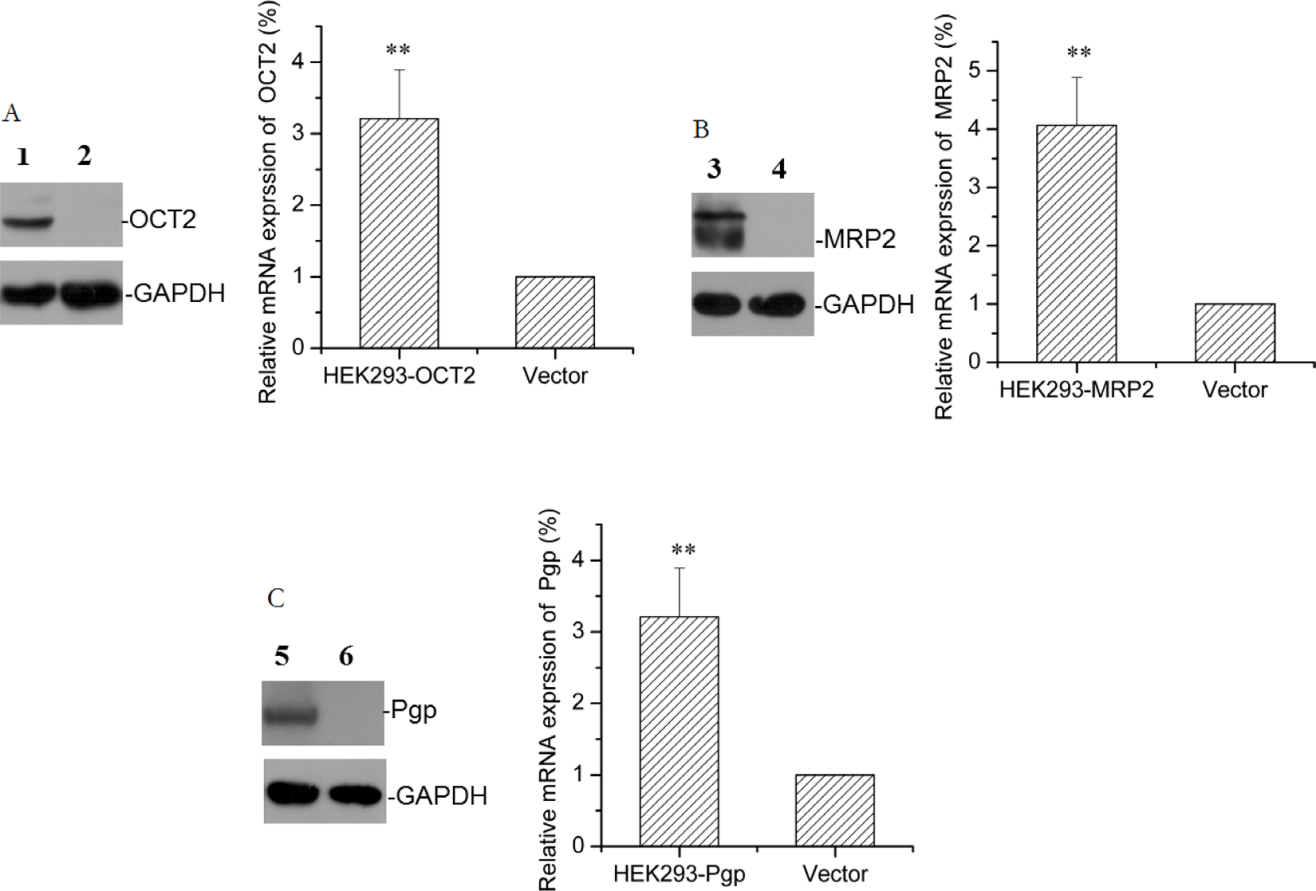
Protein and mRNA expression in transfected cells The representative protein bands and the relative mRNA levels of OCT2 **(A)**, Mrp2 **(B)**, and Pgp **(C)** in HEK293 transfected cells overexpressing OCT2, Mrp2, and Pgp or in HEK293-vector cells are shown. (1) HEK293-OCT2, (3) HEK293-Mrp2, (5) HEK293-Pgp; (2, 4, 6) HEK293-vector. Error bars indicate SD. **p < 0.01 compared with the vector.

After screening G418 for 8 weeks, transfection efficiency of HEK293-OCT2, HEK293-Mrp2, HEK293-Pgp, and HEK293-vector cells (transfected with pCMV6-AC-GFP plasmid cells) were measured with flow cytometry (Figure 2) and the data were as follows: 76%, 69%, 66%, and 82%. Western blot confirmed that stable transfected HEK293-OCT2, HEK293-Mrp2, and HEK293-Pgp cells overexpressed OCT2, Mrp2, and Pgp proteins, and vector cells hardly expressed OCT2, Mrp2, or Pgp proteins. RT-qPCR data confirmed that compared to the vector cells, OCT2, Mrp2, and Pgp mRNA expression in HEK293-OCT2, HEK293-Mrp2, and HEK293-Pgp cells was increased by 3.21, 4.07, and 2.99 times, respectively (Figure 3). Thus, HEK293-OCT2, HEK293-Mrp2, and HEK293-Pgp cells were stably transfected.

### Effect of clerosterol on activity and Oct2 and Mrp2 in BRL cell protein and mRNA expression

**Figure 4:**
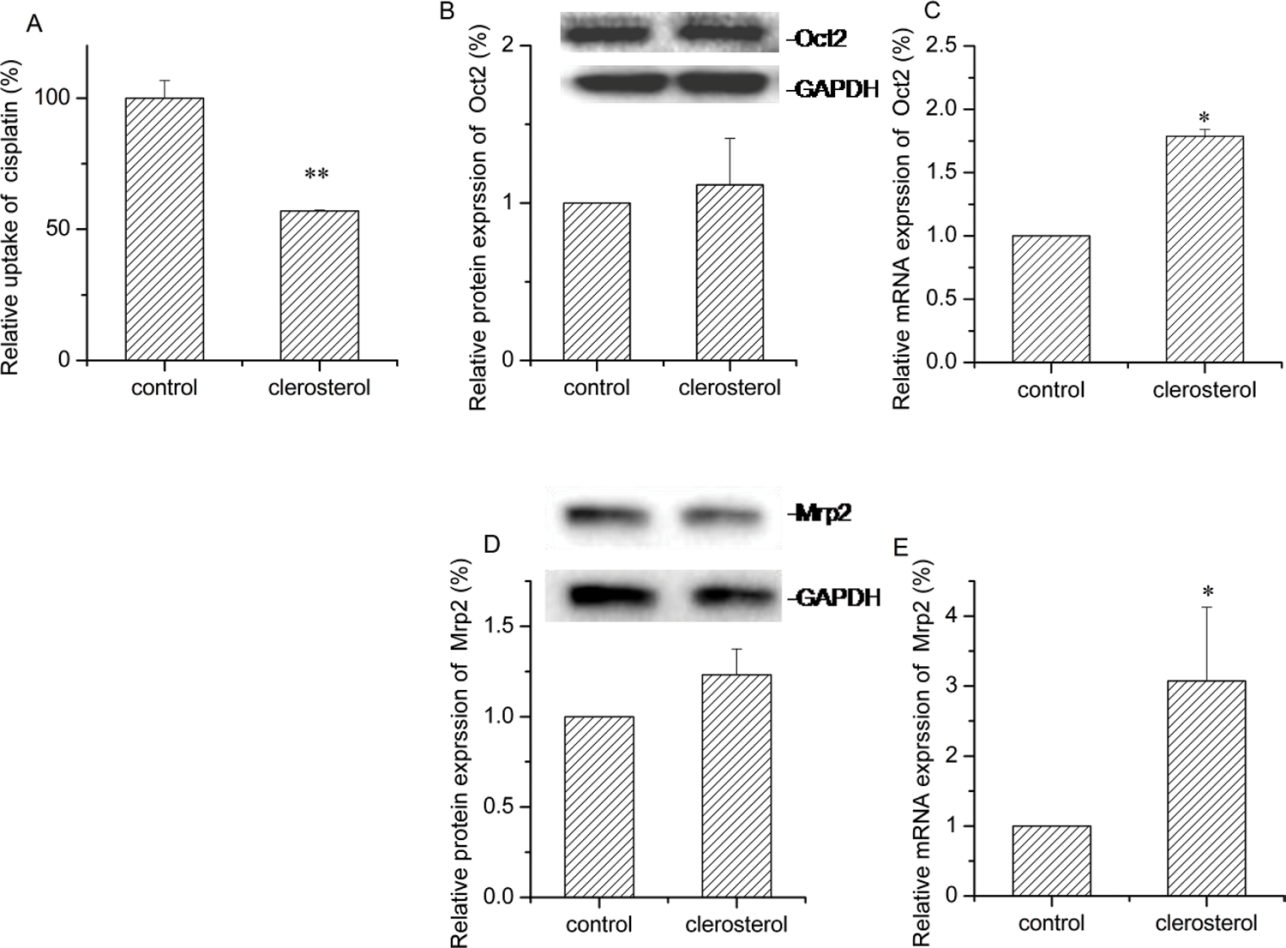
Effect of clerosterol on Mrp2 and Oct2 in BRL cells **(A)** Cisplatin uptake (DDP) was analyzed by HPLC after the BRL cells were incubated with clerosterol for 24 h at 37°C, and then incubated with 50 μg/mL of cisplatin for another 4 h at 37°C. Oct2 protein expression **(B)** and Mrp2 protein expression **(D)** in the BRL cells treated with clerosterol for the indicated period of time, determined by Western blot analysis. GAPDH was used as a loading control. Oct2 mRNA expression **(C)** and Mrp2 mRNA expression **(E)** was measured by RT-qPCR analysis. Error bars indicate SD. *p < 0.05, **p < 0.01 compared with the control.

After clerosterol treatment (24.3 μM) of BRL cells for 24 h, cisplatin uptake decreased by 53.0% (P < 0.05) (Figure 4A). Western blot data confirmed that clerosterol increased protein expression Oct2 by 11.6% (Figure 4B) and Mrp2 by 23.2% (Figure 4D). RT-qPCR data show that clerosterol increased mRNA expression of Oct2 by 78.7% (P < 0.05) (Figure 4C) and Mrp2 by 207.5% (P < 0.01) (Figure 4E). Thus, clerosterol decreased cisplatin uptake via increased Mrp2 expression.

### Effect of clerosterol on Pgp and Mrp1 activity, mRNA, and protein in HEK293 cells

**Figure 5:**
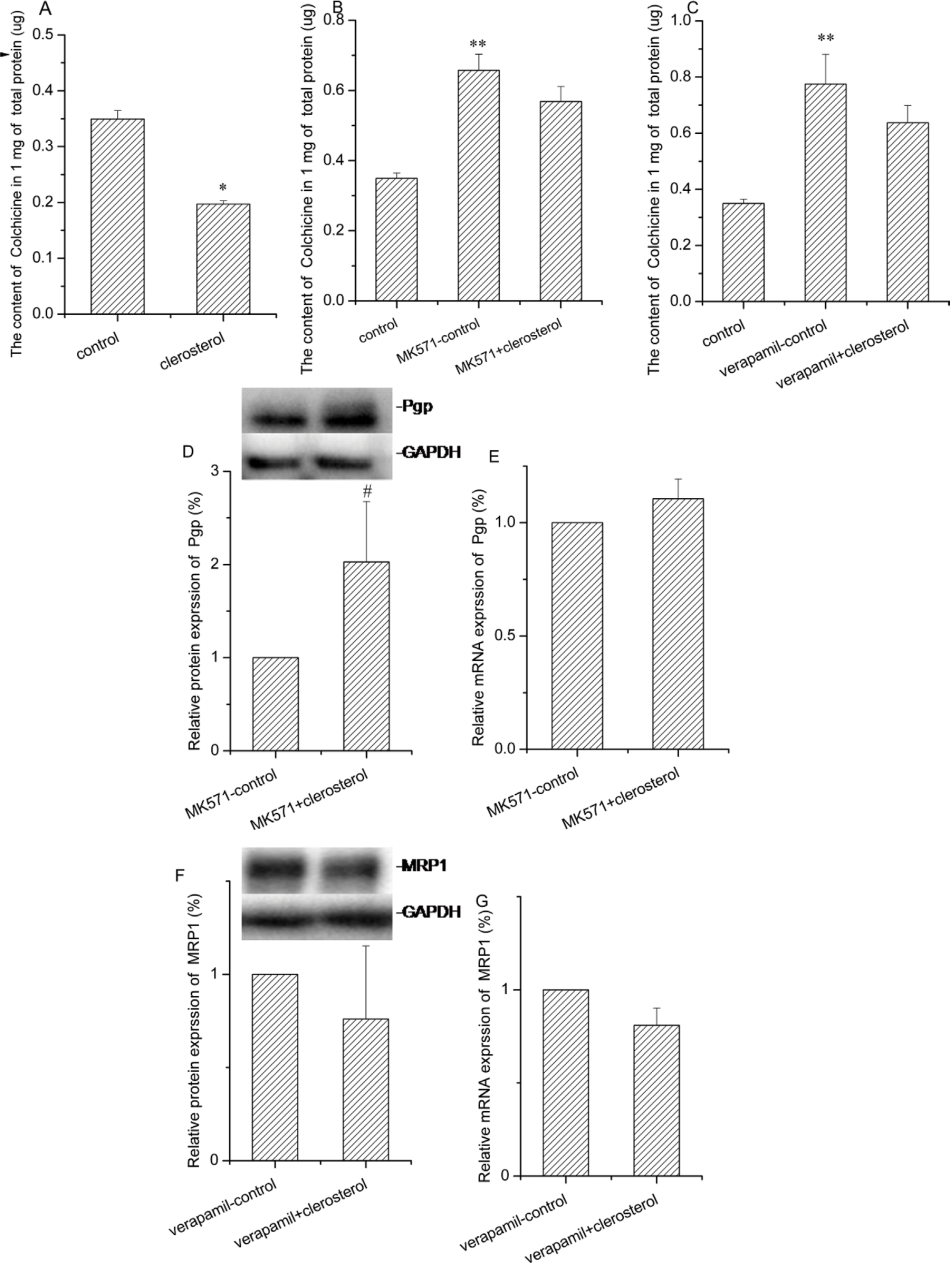
Clerosterol's effect on Mrp1 and Pgp in HEK293 cells **(A)** Colchicine uptake was analyzed by HPLC after the HEK293 cells were incubated with clerosterol for 48 h and then incubated with 50 μM of colchicine for another 1 h. **(B)** Colchicine uptake was analyzed in HEK293 cells treated with MK571 and MK571 plus clerosterol for 48 h and then incubated with 50 μM of colchicine for 1 more h. **(C)** Colchicine uptake was analyzed in HEK293 cells treated with verapamil and verapamil plus clerosterol for 48 h, and then incubated with 50 μM of colchicine for 1 more h. Pgp protein expression in HEK293 cells treated with MK571 and MK571 plus clerosterol **(D)**, and Mrp1 protein expression in the HEK293 cells treated with verapamil and verapamil plus clerosterol for 48 h **(F)** were determined by Western blot analysis. GAPDH was used as a loading control. Pgp mRNA expression **(E)** and Mrp1 mRNA expression **(G)** were measured by RT-qPCR analysis. Error bars indicate SD. *p < 0.05, **p < 0.01 compared with the control, # *p* < 0.05 compared with the MK571-control.

Treatment with clerosterol (121.5 μM) for 48 h decreased colchicine uptake by 43.6% (P < 0.01) (Figure 5A). In order to distinguish the contribution of Pgp or MRP1 on colchicine uptake, we used MK571 or verapamil as Mrp1 or Pgp inhibitors, respectively. For 48 h in HEK293 cells, colchicine uptake increased in both suggesting that both transporters contributed to colchicine efflux (the second bar of Figure 5B and 5C). Clerosterol had no significant effect on colchicine uptake when compared to the MK571-control group or verapamil-control group, but showed a tendency of decreasing colchicine uptake (the third bar of Figure 5B and 5C). These indicated that colchicine uptake decrease is based on the co-effect of promoting both Pgp and MRP1 activity. Clerosterol increased Pgp protein expression by 102.8% (P < 0.05) (Figure 5D) and had no significant effect on Mrp1 protein expression (Figure 5F). RT-qPCR data suggested that either of these changes were not biologically significant (Figure 5E and 5G). Thus, our data suggested that clerosterol increased Pgp activity by altering Pgp protein expression, but for Mrp1, not through translational regulation.

### Clerosterol increased OCT2 activity in HEK293-OCT2 cells

**Figure 6:**
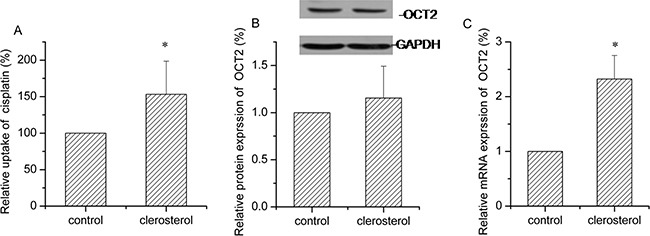
Clerosterol affects OCT2 activity in the HEK293-OCT2 cells **(A)** Cisplatin uptake was analyzed by HPLC after the HEK293-OCT2 cells were incubated with clerosterol for 24 h at 37°C, and then incubated with 50 μg/mL of cisplatin for another 4 h at 37°C. **(B)** OCT2 protein expression in the HEK293-OCT2 cells treated with clerosterol for the indicated period of time, determined by Western blot analysis. GAPDH was used as a loading control. **(C)** OCT2 mRNA expression was measured by RT-qPCR analysis. Error bars indicate SD. *p < 0.05, **p < 0.01 compared with the control.

Transporter expression is typically enhanced or inhibited in disease states, so we measured the effects of clerosterol on OCT2 activity in HEK293-OCT2 cells and noted that after 24 h, clerosterol (121.5 μM) treatment increased cisplatin uptake by 53.2% (P < 0.05) (Figure 6A). Western blot confirmed that clerosterol tended to increase OCT2 protein expression (Figure 6B) and RT-qPCR showed that OCT2 mRNA expression was increased by 132.4% (P < 0.05) (Figure 6C).

### Clerosterol had marginal effects on MRP2 activity in HEK293- MRP2 cells

**Figure 7:**
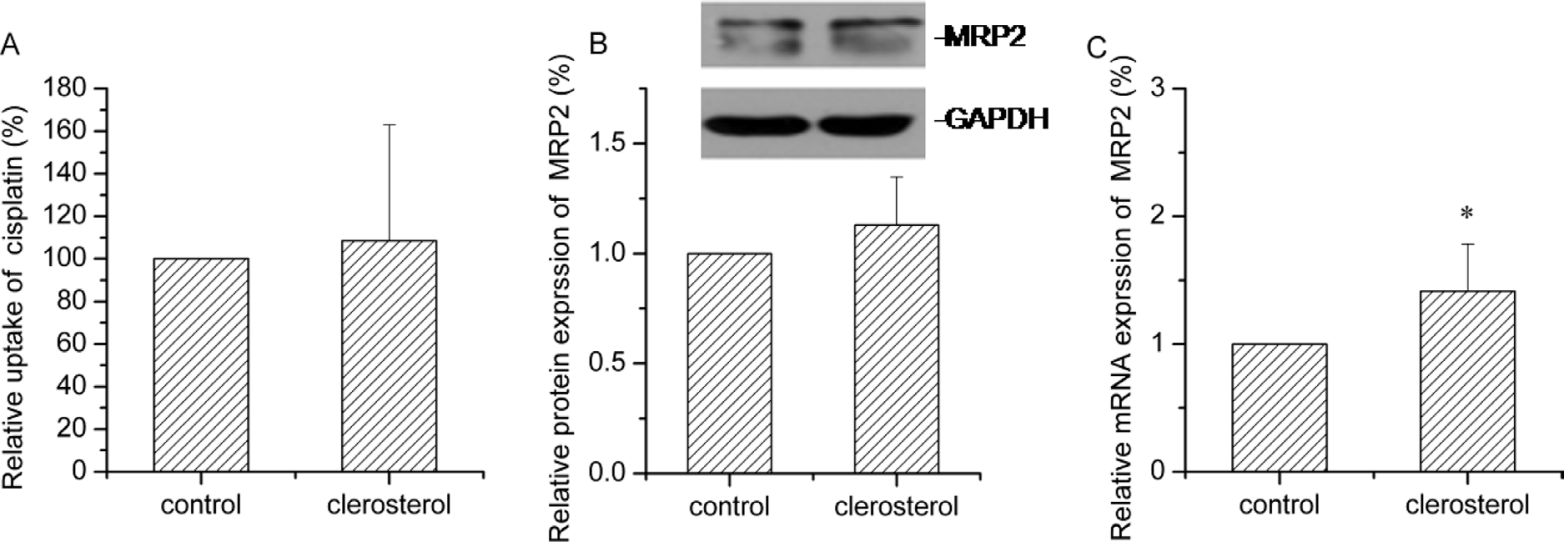
Clerosterol's effect on MRP2 in the HEK293- MRP2 cells **(A)** Cisplatin uptake was analyzed by HPLC after the HEK293-MRP2 cells were incubated with clerosterol for 24 h at 37°C, and then incubated with 50 μg/mL of cisplatin for another 4 h at 37°C. **(B)** MRP2 protein expression in the HEK293-OCT2 cells treated with clerosterol for the indicated period of time, determined by Western blot analysis. GAPDH was used as a loading control. **(C)** MRP2 mRNA expression was measured by RT-qPCR analysis. Error bars indicate SD. *p < 0.05 compared with the control.

Clerosterol treatment of HEK293-MRP2 cells for 24 h produced no significant effect on cisplatin uptake (Figure 7A). MRP2 gene and protein data were in agreement with these findings (Figure 7B and 7C).

### Clerosterol inhibited Pgp activity in HEK293-Pgp cells

**Figure 8:**
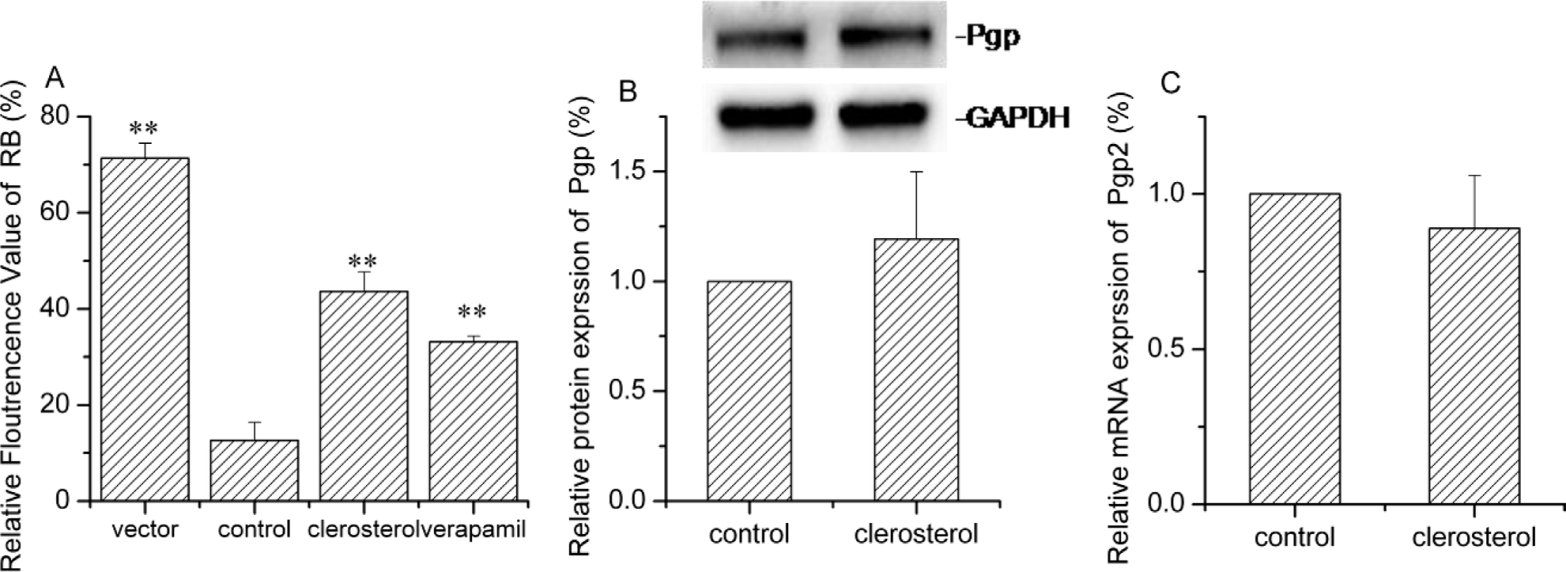
Clerosterol's effect on Pgp in the HEK293-Pgp cells **(A)** Rhodamine B uptake was determined by flow cytometry after the HEK293-Pgp cells were incubated with clerosterol or verapamil for 24 h, and then incubated with rhodamine B for another 30 min. **(B)** Pgp protein expression in the HEK293-Pgp cells treated with clerosterol for the indicated period of time, determined by Western blot analysis. GAPDH was used as a loading control. **(C)**. Pgp mRNA expression was measured by RT-qPCR analysis. Error bars indicate SD. *p < 0.05, **p < 0.01 compared with the control.

Figure 8A shows reduced rhodamine B (Pgp substrate) uptake after Pgp transfection. Clerosterol or verapamil (Pgp inhibitor) treatment of transfected cells for 24 h caused increased rhodamine B uptake by 31.0% and 20.5% (P < 0.01). Western blot and RT- q PCR data indicated that clerosterol had no effect on Pgp protein and mRNA expression in HEK293-Pgp cells after 24 h compared to the controls (Figure 8B and 8C). Thus, Clerosterol suppressed Pgp overexpression but not by regulating gene expression.

### Clerosterol and MRP1 activity in glutathione-stimulated HEK293 cells

**Figure 9:**
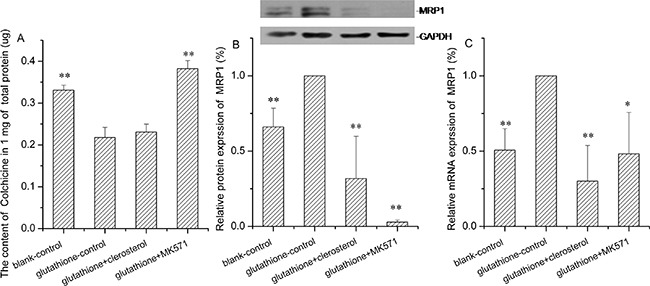
Clerosterol's effect on MRP1 in glutathione-stimulated HEK293 cells **(A)** Colchicine uptake was determined by HPLC after the HEK293 cells were incubated with glutathione, glutathione plus clerosterol, and glutathione plus MK571 for 24 h, and then incubated with Colchicine for another 1 h. **(B)** MRP1 protein expression in HEK293-Pgp cells treated with glutathione, glutathione plus clerosterol and glutathione plus MK571 for the indicated period of time, determined by Western blot analysis. GAPDH was used as a loading control. **(C)** Pgp mRNA expression was measured by RT-qPCR analysis. Error bars indicate SD. *p < 0.05, **p < 0.01 compared with the glutathione-control.

MRP1 is a glutathione-dependent protein [16], which increases activity with increasing glutathione. In HEK293 cells stimulated with glutathione, colchicine uptake decreased and Mrp1 protein and gene expression increased significantly compared to the controls (the second bar of Figure 9A, 9B, and 9C). After MK571 (Mrp1 inhibitor) treatment, colchicine increased and Mrp1 protein and gene expression decreased compared with the glutathione controls (the forth bar of Figure 9A, 9B, and 9C). Thus, the Mrp1 activity enhancing model was successful. Clerosterol did not change colchicine uptake in glutathione-stimulated HEK293 cells, but it decreased protein and mRNA expression compared to the glutathione controls (the third bar of Figure 9A, 9B, and 9C). These results indicated that clerosterol affected the protein and mRNA expression of MRP1, but it did not affect MRP1 function.

## DISCUSSION

VBRB is usually used in liver cancer therapy as a complement to and combined with chemtoxicity drugs, and is belived to have synergestic effects. Since VBRB is usually used as powder directly, we hypothesized that there are some hydrophilic components in VBRB active at the VBRB-induced herb-drug interaction, therefore, we investigated ethyl acetate parts of VBRB, and clerosterol was found in VBRB for the first time.

Drug transporters are critical for therapeutic efficacy and toxicity as well as herb-drug interactions [7, 17, 18]. VBRB may improve chemotherapeutic efficacy and decrease toxicity and these actions may occur via drug transporters. Therefore, the effect of clerosterol on transporters in normal and disease states was studied.

Cisplatin was used as both an Oct2 and Mrp2 substrate. Increased Oct2 transport activity increased cisplatin uptake and increased Mrp2 transport activity promoted cisplatin efflux, so we studied the effect of clerosterol on the Oct2 and Mrp2 activity in normal BRL cells by measuring cisplatin accumulation. Our results showed clerosterol decreased cisplatin uptake. In further protein and mRNA studies, we confirmed that this effect is mainly through increasing Mrp2 gene expression. Colchicine is a substrate for Pgp and Mrp1 so the effects of clerosterol on Pgp and Mrp1 activity in normal HEK 293 cells were studied with colchicine as a probe. Clerosterol decreased colchicine uptake, and this effect was co-contributed by both Pgp and Mrp1. Cisplatin and colchicine are common chemotherapy medicines, but both are limited by toxicity and resistance. Our results suggested that clerosterol might decrease the toxicity of cisplatin and colchicines in normal cells by inhibiting their uptake.

Multidrug resistance is an important problem in chemotherapy. Overexpressing multidrug resistance transporters is the main mechanism of drug resistance [19, 20, 21] so we next studied the clerosterol effect on transporters in transporter-overexpressing HEK293 cells.

Clerosterol inhibited Pgp expression in HEK293-Pgp cells significantly when Pgp is overexpressed in cancer cells, giving rise to multi-drug resistance and chemotherapeutic failure. Pgp is reported to be found in most organs, but its hepatic expression is in abundant, suggesting that clerosterol may safely inhibit hepatic Pgp [18].

MRPs are transporters that normally protect tissues against exogenous compounds, and in disease states may cause therapeutic failure [22]. Clerosterol's effect on Mrp1 and Mrp2 differs: clerosterol had marginal effects on normal or enhanced Mrp1 activity and clerosterol enhanced typical Mrp2 in BRL 3A cells, but had marginal effects on overexpressed Mrp2 in HEK 293 cells. Thus, clerosterol may not be useful for MRP-induced multi-drug resistance. Mrp2 contributes to bile acid and bilirubin transport, and decreased Mrp2 activity causes cholestasis, which may be amenable to clerosterol treatment by increasing Mrp2 activity.

OCT2 is an influx transporter that moves cationic compounds and clerosterol enhanced OCT2 activity in physilogical and pathological states. OCT2 contributes to renal toxicity and ototoxicity of cisplatin [23, 24], so clerosterol should likely not be co-administered with cisplatin.

Collectively, clerosterol isolated from VBRB may be an active constituent of the herb and may work against cancer multidrug resistance by inhibiting the activity of multidrug resistance transporters, especially Pgp.

## MATERIALS AND METHODS

### Plant materials and clerosterol isolation

Decoction pieces of VBRB were purchased from Kangmei Medical Company (Guangzhou, China), and authenticated by Professor Rhizhi, Zhao (Second Affiliated Hospital, Guangzhou University of Chinese Medicine). A voucher sample (120100341) was deposited in the Laboratory of Chinese Medicine Preparation, at the Second Affiliated Hospital, Guangzhou University of Chinese Medicine. Decoction pieces (10 kg) were extracted with 80% ethanol 3 times (2 h each time). The ethanol extract was filtered and concentrated using a rotary evaporator. Crude extract was dissolved with ethyl acetate (3,000 mL) and concentrated. The ethyl acetate soluble fraction (178.6 g) was dissolved in ethyl acetate and subjected to silica gel column chromatography using petroleum ether-ethyl acetate (20:1-1:1) as the eluent. The sub-fraction (petroleum ether-ethyl acetate, 10:1) yielded a white crystal. Final purification was accomplished by recrystallization with ethyl acetate to afford a white needle-like crystal (compound 1, Figure 1A). The structure of compound 1 was elucidated using NMR and MS. Compound 1 (>98%) was evaluated with HPLC.

### Materials

An MTT kit was purchased from Sigma-Aldrich (St. Louis, MO). Fetal bovine serum (FBS), Dulbecco's modified Eagle's medium (DMEM) in high glucose, penicillin-streptomycin solution (100×), 0.25% Trypsin-EDTA, and PBS were acquired from Gibco Company (Grand Island, NY). DMSO was bought from Guangzhou Chemical Reagent Factory (Guanzhou, China). OCT2, Pgp, Mrp1, and Mrp2 mouse monoclonal antibodies and anti-mouse IgG HRP-conjugated antibody were purchased from Abcam (Cambridge, UK). GAPDH rabbit monoclonal antibody and anti-rabbit IgG HRP-conjugated antibody were obtained from Cell Signaling Technology (Beverly, MA). OCT2-pCMV6-AC-GFP, Mrp2-pCMV6-AC-GFP, ABCB1-pCMV6-AC-GFP, and pCMV6-AC-GFP plasmids and Mega Tran 1.0 transfection reagent were purchased from OriGene Technologies Company (USA). Aminoglycoside antibiotic (G418) was purchased from Guangzhou Xueyou Biotech Limited Company (Guanzhou, China).

### Cell lines and cell culture

A BRL normal rat hepatocyte cell line and a HEK293 human embryonic kidney cell line were obtained from American Type Culture Collection (ATCC, USA). BRL cells were maintained in DMEM medium supplemented with 5% FBS, penicillin (100 IU/mL), and streptomycin (100 mg/mL). HEK293 cells were maintained in DMEM medium containing 10% FBS, penicillin (100 IU/mL), and streptomycin (100 mg/mL), and the glutathione-stimulated HEK293 cell model contained glutathione (2 mM). Cells were incubated at 37°C in a humidified atmosphere containing 5% CO_2_ and 95% air.

### Viability assays

Cell viability was measured using MTT assay. Briefly, BRL and HEK 293 cells were seeded onto 96-well plates (2 × 10^3^ cells/well and 4 × 10^3^ cells/well^−1^, respectively). After incubation for 24 h at 37°C and 5% CO_2_, 150 μL of clerosterol solution (243.0, 121.5, 48.6, 24.3, 12.1, 2.43, and 0.24 μM) was added and co-cultured for 48 h. Then, 20 μL of MTT (50 g/L) was added to each well for another 4 h and formazan formation and dissolution in 150 μL of DMSO were performed. Optical density was measured at 570 nm and control optical density was considered 100% viability.

### Stable HEK293 cells overexpressing OCT2, Mrp2, and Pgp selection

HEK293 cells were seeded in 6-well plates and grown to 80% confluency, then OCT2-pCMV6-AC-GFP, Mrp2-pCMV6-AC-GFP, ABCB1-pCMV6-AC-GFP, and pCMV6-AC-GFP plasmids were transfected with plasmid DNA with a green fluorescent protein reporter gene. Genes were introduced into HEK293 cells with MegaTran 1.0. 72 h after transfection, and HEK293 cells overexpressing OCT2, Mrp2, and Pgp (HEK293-OCT2, HEK293-Mrp2, and HEK293-Pgp) were established with aminoglycoside selection (G418, 578 μM) for 8 weeks. Transfection efficiency was confirmed with flow cytometry, and Western blot and RT-qPCR were used to measure protein and mRNA, respectively.

### Cisplatin uptake assay

Cisplatin is a co-substrate of OCT2 and Mrp2 and cisplatin uptake was expressed as cisplatin/mg protein. Cells were seeded into 6-well plates and incubated at 37°C until reaching a logarithmic growth phase. Then, clerosterol (24.3 μM in BRL cells, 121.5 μM in HEK293 -OCT2 or -Mrp2 cells) was added for 24 h. Subsequently, cells were treated with 50 μg/mL of cisplatin for another 4 h and harvested. After freezing and thawing harvested cells 3 times with cell lysate solution (Tris 100 mM, EDTA 5 mM, NaCl 200 mM, SDS 0.2%, at pH 8), cell lysate was centrifuged and supernatant was collected. A 175 μL sample after ultrafiltration or a 175 μL of cisplatin standard solution was obtained and 10 μg of NiCl_2_ and 20 μL of DDTC solution (0.25 M) were added to each. The mixture was vortexed for 1 min and incubated at 37°C in a water bath for 15 min. Finally, 175 μL of chloroform was added and the samples were centrifuged for 5 min after vortexing for 1 min. The final solution was injected (10 μL volume) into an HPLC system. Separation was conducted using a Diamonsil C18 column (250 × 4.6 mm, 5 μm) with a water-methanol-acetonitrile (23:46:31) mobile phase with a flow rate of 1.5 mL·min^−1^. The column temperature was kept at 30°C and 254 nm was the selected wavelength. Cisplatin uptake was expressed as cisplatin/mg protein.

### Colchicine uptake assay

Cells were seeded into 6-well plates and incubated at 37°C until reaching a logarithmic growth phase. Clerosterol and MK571 or verapamil were added and co-cultured for 48 h. Then, cells were treated with 50 μM of colchicine for another 60 min and harvested. After freezing and thawing harvested cells with redistilled water and centrifuging at 15,000 x *g* for 15 min, the supernatant was collected. Then, 175 μL of sample was added with 3 volume of methanol and vortexed for 1 min. The supernatant was transferred to a clean tube and evaporated to dryness after centrifugation at 15,000 × *g* for 15 min. The obtained residue was dissolved in 175 μL of mobile phase and centrifuged at 15,000 × *g* for another 15 min. The supernatant was taken and injected (25 μL volume) into HPLC. Separation was conducted using a Diamonsil C18 column (250 × 4.6 mm, 5 μm). The mobile phase was water-methanol (45:55) with a flow rate of 1mL·min^−1^. The column temperature was kept at 25°C and the detection wavelength was 353 nm. Colchicine uptake was expressed as colchicine/mg protein.

### Rhodamine B uptake assay

Fluorescent intensity of intracellular rhodamine B was measured with flow cytometry. Cells at a logarithmic growth phase were treated with clerosterol in 6-well plates for 24 h, then the supernatant was removed and the cells were washed with PBS. Subsequently, rhodamine B was added. After being co-cultured in the dark for 30 min, cells were harvested and washed with PBS. Relative fluorescence of intracellular rhodamine B was quantified with flow cytometry (10,000 cells/experiment) for 3 independent experiments.

### Western blot analysis

Cells were treated with clerosterol for 24 h and harvesting, then lysed in RIPA cell buffer with protease inhibitor on ice and vortexed 10 min, 3 times each. Then cell lysates were centrifuged at 15,000 x g for 15 min and supernatant was collected for protein measurement. Protein samples were boiled in loading buffer for 5 min, and separated by SDS PAGE. Proteins were transferred to PVDF membranes at 100 V for 2 h, and membranes were blocked for 1 h in TBS containing 0.1% Tween 20 and 5% (w/v) dry skimmed milk powder. Membranes were incubated overnight at 4°C with primary antibodies to OCT2 (1:500), Mrp1 (1:1,000), Mrp2 (1:500), or Pgp (1:1,000). Membranes were washed with TBS containing 0.1% Tween 20 and incubated for 1 h with secondary antibody. Protein bands were developed with ECL and exposed on X-ray film. Developed blots were scanned and quantified using a Gel Doc XR Image Analyzer (Bio-Rad Laboratories, Berkeley, CA). Densitometric assessment of the target protein on blots compared to the reference proteins was used to quantify the protein.

### Quantitative real-time PCR experiments

Cells were treated with clerosterol for 24 h, and then the total RNA was extracted with Trizol reagent (Invitrogen, Life Technologies, Carlsbad, CA) according to the manufacturer's instructions. 1 μg of total RNA was used as a template for each reverse transcriptase (RT)-mediated PCR using a RevertAid First Strand cDNA synthesis kit (Thermo Fisher Scientific, Waltham, MA), as described in the manufacturer's instructions. Real-time PCR was performed in 96-well plates using an ABI 7500 real-time PCR machine (Applied Biosystems, UK). Primers were as follows: rat Oct2, forward 5′-CCGAGAATATGCAGAGGCCAA-3′ and reverse 5′-AAGTCAGCTCCAGCAGCA

AT-3′; rat Mrp2, forward 5′-CCCGCCAGCT GAGACGGTTG-3′ and reverse 5′-GCTGGTGCTCA AAGGCACGGA-3′; rat gapdh, forward 5′-ATGAT TCTACCCA

CGGCAAG-3′ and reverse 5′-CTGGAAGAT GGTGATGGGTT-3′; human OCT2, forward 5′-TGCAGC TGGAGTTCTCATGG-3′ and reverse 5′-CTCCGA TATCTCCGCC

CAAC-3′; human Mrp1, forward 5′-gggggagaa aaggtcggcatcg-3′ and reverse 5′-GTGCAGGCCG ATCTTGGCGA-3′; human Mrp2, forward5′-ACAG TCCGA

GATGTGAACCTG-3′ and reverse 5′-TGAATC CAGGACTGCTGTGG-3′; human Pgp, forward 5′-AC TTGTCACAATGCAGACAGCAGG-3′ and reverse 5′-TGTGATCCACG GACACTCCTACG-3′; and human GAPDH, forward 5′-GATCATCAGCAAT GCCTCCTGCACC-3′ and reverse 5′-ACTTGT CACAATGCAGACAGCAGG-3′. Amplification reactions were performed as follows: 10 min at 95°C, and 40 cycles of 95°C for 15 s and 60°C for 1 min. Data obtained were analyzed with the Relative Quantification (ddCt) Study program.

### Statistical analysis

All experiments represented in Figures 4, 5, 6, 7, 8, 9 were repeated at least 3 times. All statistical analyses were performed using SPSS 17.0. Data are means ± SD. Statistical differences for pairwise comparisons were determined using an independent-samples *t* test. For multiple comparisons, analysis was performed with Fishers Least Significant Difference (LSD) test, and Dunnett's T3 was used for variance heterogeneity (*p* < 0.05 or *p* < 0.01 was regarded as statistically significant).

## References

[1] Kulik LM, Chokechanachaisakul A (2015). Evaluation and management of hepatocellular carcinoma. Clinic Liver Disease.

[2] Wan YH, Liu LH, Huang WQ (2013). Clinical observation on integrated Chinese and western treatment on 74 cases of primary liver cancer. Chinese Medicine Modern Distance Education of China.

[3] Zhang TS, Shang GZ (2011). Clinical observation on integrated Chinese and western treatment on 46 cases of primary liver cancer. Jiangxi Zhong Yi Yao.

[4] Liu YK, Gong CC, Xie L (2007). Effect of modified xiao chaihu tang on protein dexpressions of apoptosis due to cisplation in rat liver cancer CBRH7919 cells. Chinese Journal of Experimental Traditional Medical Formulae.

[5] Wang SL, Wang Y, Zhao JR, Zhao HX, Li BB, Song W, Liu CY (2011). >The Experimental Research of Small Entrapement Soup Combined Cyclophosphamide Affect EAC Hepatumor Cell Apoptosis in Mice. Journal of Practical Chinese Internal Medicine.

[6] Dlugosz A, Janecka A (2016). ABC transporters in the development of multidrug resistance in cancer therapy. Current Pharmaceutic Design.

[7] Schinkel AH, Jonker JW (2003). Mammalian drug efflux transporters of the ATP binding cassette (ABC) family: an overview. Advanced Drug Delivery Reviews.

[8] Gai XD, Zeng CQ, Hong M (2005). Ef fect of Bupleurun Chinese DC(BCDC) on the MDR Reversal of Hepatocel lular Carcinoma and Related Mechanism. Chemical Journal of Chinese Universities.

[9] Zhao R, Liu L, Wang Y, Xiao Z (2014). Vinegar-baked Radix Bupleuri modulates the cell membrane constituents and inhibits the P-gp activity in rat hepatocytes. BMC Complementary Alternative Medicine.

[10] Ciarimboli G (2014). Membrane transporters as mediators of cisplatin side-effects. Anticancer Research.

[11] Sprowl JA, Lancaster CS, Pabla N, Hermann E, Kosloske AM, Gibson AA, Li L, Zeeh D, Schlatter E, Janke LJ, Ciarimboli G, Sparreboom A (2014). Cisplatin-induced renal injury is independently mediated by OCT2 and p53. Clinic Cancer Research.

[12] Bachran C, Bachran S, Sutherland M, Bachran D, Fuchs H (2008). Saponins in tumor therapy. Mini- Review Medicinal Chemistry.

[13] Lu XL, He SX, Ren MD, Wang YL, Zhang YX, Liu EQ (2011). Chemopreventive effect of saikosaponin-d on diethylinitrosamine-induced hepatocarcinogenesis: involvement of CCAAT/enhancer binding protein beta and cyclooxygenase-2. Molecular Medicine Reports.

[14] Wong VK, Zhang MM, Zhou H, Lam KY, Chan PL, Law CK, Yue PY, Liu L (2013). Saikosaponin-d Enhances the Anticancer Potency of TNF-alpha via Overcoming Its Undesirable Response of Activating NF-Kappa B Signalling in Cancer Cells. Evidence Based Complementary Alternative Medicine.

[15] Kwon HC, Min YD, Kim KR, Bang EJ, Lee CS, Lee KR (2003). A new acylglycosyl sterol from Quisqualis Fructus. Archives Pharmacal Research.

[16] Renes J, de Vries EG, Nienhuis EF, Jansen PL, Muller M (1999). ATP- and glutathione-dependent transport of chemotherapeutic drugs by the multidrug resistance protein MRP1. British Journal of Pharmacology.

[17] DeGorter MK, Xia CQ, Yang JJ, Kim RB (2011). Drug transporters in drug efficacy and toxicity. Annual Review Pharmacology and Toxicology.

[18] Szakacs G, Varadi A, Ozvegy-Laczka C, Sarkadi B (2008). The role of ABC transporters in drug absorption, distribution, metabolism, excretion and toxicity (ADME-Tox). Drug Discovery Today.

[19] Dury L, Nasr R, Lorendeau D, Comsa E, Wong I, Zhu X, Chan KF, Chan TH, Chow L, Falson P, Di Pietro A, Baubichon-Cortay H (2017). Flavonoid dimers are highly potent killers of multidrug resistant cancer cells overexpressing MRP1. Biochemical Pharmacology.

[20] Wu T, Chen Z, To KK, Fang X, Wang F, Cheng B, Fu L (2017). Effect of abemaciclib (LY2835219) on enhancement of chemotherapeutic agents in ABCB1 and ABCG2 overexpressing cells in vitro and in vivo. Biochemical Pharmacology.

[21] Chen Z, Chen Y, Xu M, Chen L, Zhang X, To KK, Zhao H, Wang F, Xia Z, Chen X, Fu L (2016). Osimertinib (AZD9291) Enhanced the Efficacy of Chemotherapeutic Agents in ABCB1- and ABCG2-Overexpressing Cells In Vitro, In Vivo, and Ex Vivo. Molecular cancer therapeutics.

[22] Zhang YK, Wang YJ, Gupta P, Chen ZS (2015). Multidrug Resistance Proteins (MRPs) and Cancer Therapy. The AAPS Journal.

[23] Ciarimboli G, Deuster D, Knief A, Sperling M, Holtkamp M, Edemir B, Pavenstädt H, Lanvers-Kaminsky C, A am Zehnhoff-Dinnesen, Schinkel AH, Koepsell H, Jürgens H, Schlatter E (2010). Organic cation transporter 2 mediates cisplatin-induced oto- and nephrotoxicity and is a target for protective interventions. The American Journal of Pathology.

[24] Filipski KK, Mathijssen RH, Mikkelsen TS, Schinkel AH, Sparreboom A (2009). Contribution of organic cation transporter 2 (OCT2) to cisplatin-induced nephrotoxicity. Clinic Pharmacology and therapeutics.

